# Association between admission hemoglobin-to-red blood cell distribution width ratio and stroke-associated pneumonia risk in acute ischemic stroke: effect modification by diabetes status

**DOI:** 10.3389/fendo.2026.1778191

**Published:** 2026-03-30

**Authors:** Hui Du, Xiaoming Su, Qianhua Huang, Wei Yu, Qunhui Huang, Xiaoqiang Li

**Affiliations:** 1 Xiaolan Clinical Institute of Shantou University Medical College, Zhongshan, China; 2 Department of Blood Transfusion, Xiaolan People’s Hospital of Zhongshan (The Fifth People’s Hospital of ZhongShan), Zhongshan, Guangdong, China; 3 Department of Laboratory Medicine, Xiaolan People’s Hospital of Zhongshan (The Fifth People’s Hospital of ZhongShan), Zhongshan, Guangdong, China; 4 Department of Neurology, Xiaolan People’s Hospital of Zhongshan (The Fifth People’s Hospital of ZhongShan), Zhongshan, Guangdong, China

**Keywords:** acute ischemic stroke, stroke-associated pneumonia, hemoglobin-to-red blood cell distribution width ratio, diabetes mellitus, retrospective cohort

## Abstract

**Objective:**

This study aimed to assess the relationship between admission hemoglobin-to-red blood cell distribution width ratio (HRR) and stroke-associated pneumonia (SAP) risk in patients with acute ischemic stroke (AIS), and to explore whether this association is modified by diabetes status.

**Methods:**

In this retrospective study of 1051 patients with AIS admitted within 72 hours of symptom onset, we first visualized the shape of the association between admission HRR and SAP risk using a generalized additive model (GAM). Subsequently, multivariable logistic regression was used to assess this association, adjusting for available confounders, yielding adjusted odds ratios (ORs) and 95% confidence intervals (CIs). The stability of this association was rigorously tested through extensive subgroup and sensitivity analyses.

**Results:**

A linear negative association was observed between HRR and SAP risk. In the fully adjusted model, HRR was inversely associated with SAP risk (adjusted OR = 0.75; 95% CI: 0.64, 0.87; p < 0.001). A significant interaction was detected between HRR and diabetes status (p for interaction = 0.01). Stratified analysis showed that the inverse association was present in non-diabetic patients (OR = 0.69; 95% CI: 0.59, 0.81; p < 0.001) but not in diabetic patients (OR = 0.97; 95% CI: 0.77, 1.21; p = 0.76).

**Conclusion:**

Admission HRR is inversely associated with SAP risk in AIS patients. This association is modified by diabetes status (p for interaction = 0.01), with the inverse association attenuated in diabetic patients. SAP risk stratification strategies may need to account for diabetes status.

## 1 Introduction

Stroke-associated pneumonia (SAP) is a common and serious complication following acute ischemic stroke (AIS), contributing to global mortality and long-term disability (1, 2). SAP worsens clinical outcomes, leading to higher mortality rates, prolonged hospital stays, and poorer functional recovery compared to patients without this complication (3–5). These consequences underscore the need for reliable methods of early risk stratification. Identifying high-risk individuals upon admission is essential for timely implementation of preventive strategies.

While risk scores like the A2DS2 and inflammatory biomarkers such as serum interleukin 6 (IL-6) and the neutrophil-to-lymphocyte ratio (NLR) have been developed to predict SAP, their clinical utility is often limited (6–8). Risk scores can be cumbersome, delaying assessment during the critical admission period (9). Conventional biomarkers typically reflect only a single dimension of SAP pathophysiology, such as the inflammatory cascade, and their levels may peak too late for early intervention (10). A novel biomarker is therefore needed—one that is readily accessible from routine admission tests and integrates multiple pathophysiological signals for more comprehensive risk assessment.

The predictive value of biomarkers may also vary across patient subgroups. Diabetes mellitus, a condition characterized by chronic inflammation and immune dysregulation, is highly prevalent in stroke patients and is associated with increased infection susceptibility (11, 12). Prior studies in cardiovascular disease have suggested that diabetes may attenuate the predictive value of inflammatory markers such as C-reactive protein, possibly due to the elevated baseline inflammatory state in diabetic patients (13). Whether diabetes status similarly modifies the association between hematological biomarkers and SAP risk remains unexplored.

Susceptibility to SAP may be determined not by a single biological pathway, but by the balance between systemic stress and physiological reserve. The complete blood count offers key indicators to assess this balance. Red blood cell distribution width (RDW) is a marker of systemic inflammation, oxidative stress, and anisocytosis, with elevated levels linked to poor outcomes in various critical illnesses (14–17). Hemoglobin (Hb) reflects systemic oxygen-carrying capacity, nutritional status, and immunocompetence, all vital for neurological recovery and infection resistance (18, 19). A patient’s vulnerability is likely maximal when high stress (high RDW) coincides with low reserve (low Hb). The Hemoglobin-to-Red Blood Cell Distribution Width Ratio (HRR) (20) is a composite index that captures this imbalance. A low HRR value represents a high-risk phenotype of depleted reserves amid elevated inflammation. This ratio-based approach has shown promise in cardiovascular diseases and cancer (21–23).

We hypothesized that HRR could serve as a useful biomarker for SAP risk. Given the distinct immunometabolic profile of diabetic patients, we also hypothesized that diabetes status may modify this association. This study aimed to investigate the association between admission HRR and early-onset SAP risk in AIS patients, with a focus on potential effect modification by diabetes status.

## 2 Methods

### 2.1 Study design and population

This retrospective cohort study utilized data from the Department of Neurology at Xiaolan People’s Hospital of Zhongshan. Individuals presenting with AIS who were hospitalized in our stroke unit between October 2019 and November 2022 were consecutively included. The institutional ethics committee of Xiaolan People’s Hospital of Zhongshan approved the study protocol (Approval No. [2025-014]), which was in accordance with the principles of the Declaration of Helsinki. Given the retrospective design of the analysis, the need for informed consent was waived by the committee.

**Figure 1:**
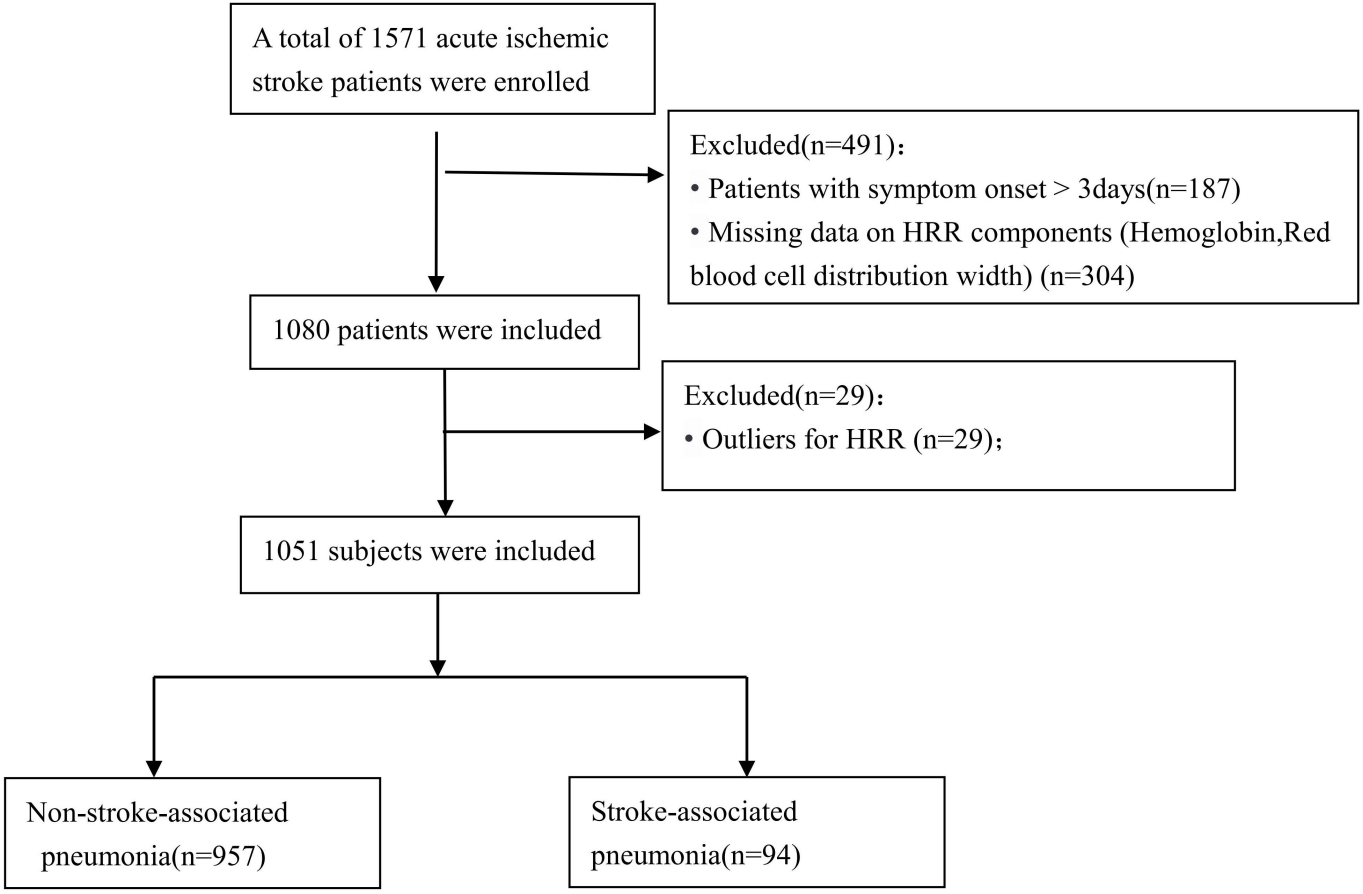
Flow chart visualizing the patient selection process.

Inclusion criteria were (1): age ≥ 18 years; (2) diagnosis of AIS confirmed by brain magnetic resonance imaging (MRI) or computed tomography (CT); (3) admission within 72 hours of symptom onset. Exclusion criteria included: (1) evidence of active infection or pneumonia on admission; (2) severe hematological disorders; (3) recent history of major surgery or trauma; (4) incomplete medical records or missing data for hemoglobin or red blood cell distribution width (RDW) from the initial blood test; and (5) outliers for HRR identified using the 1.5×IQR rule (values < 6.13 or > 15.17), resulting in exclusion of 29 patients (2.69%). A total of 1051 patients were included in the final analysis, as illustrated in **Figure 1**.

### 2.2 Data collection and definitions

We collected baseline demographic, clinical, and laboratory data from the hospital’s electronic medical records. This included age, sex, medical history (hypertension, diabetes mellitus, coronary heart disease, atrial fibrillation), and treatment details (e.g., intravenous thrombolysis). Laboratory parameters were obtained from the first blood sample drawn upon admission. HRR was calculated as the Hb (g/L) divided by the RDW value (%). The primary outcome was the development of SAP during hospitalization. SAP was diagnosed according to the 2015 consensus criteria from the Pneumonia in Stroke Consensus Group, as outlined in their recommendations for stroke-associated pneumonia diagnosis (24). A diagnosis of SAP required clinical evidence of lower respiratory tract infection, including at least one of the following: (1) fever >38 °C without other identifiable cause; (2) abnormal white blood cell count (leukocytosis or leukopenia); (3) new-onset pulmonary crackles on auscultation; or (4) new or progressive infiltrates on chest radiographs suggestive of pneumonia.

### 2.3 Statistical analysis

We presented the baseline demographic and clinical data for the entire cohort, followed by a comparison between patients with and without SAP. Continuous data were reported as mean standard deviation (SD) or median (interquartile range, IQR), while categorical data were shown as counts (percentages). To assess differences between groups, the Student’s t-test, Mann-Whitney U test, or chi-square test was applied as appropriate. Standardized Mean Differences (SMDs) were computed to quantify the balance of baseline covariates, with an SMD > 0.1 deemed a meaningful imbalance. Missing data were handled using multiple imputation by chained equations (MICE). Only three variables had missing values: mean platelet volume (MPV, 8 patients, 0.8%), plateletcrit (PCT, 8 patients, 0.8%), and platelet distribution width (PDW, 8 patients, 0.8%). Twenty imputed datasets were generated, and results were pooled using Rubin’s rules. The imputation model included all variables used in the final regression models to preserve relationships between variables. To characterize the dose-response relationship, a generalized additive model (GAM) fitted with a smoothing spline was utilized to graphically assess whether the association between continuous HRR and the likelihood of SAP was linear or non-linear. Multivariable logistic regression was then used to quantify the association between HRR and SAP risk. A series of three sequential models was developed: (1) a Crude Model (unadjusted); (2) Model 1, which adjusted for age and sex; and (3) Model 2, which further adjusted for the covariates in Model 1 plus history of hypertension, diabetes mellitus, coronary heart disease, atrial fibrillation, intravenous thrombolysis, and relevant laboratory values. In these models, HRR was evaluated both as a continuous variable (per unit increase) and as a categorical variable (by tertiles: T1, T2, T3). Odds ratios (ORs) with their 95% confidence intervals (CIs) were generated for each model. A P for trend was determined by treating the tertile categories (T1-T3) as a continuous ordinal variable (coded as 1, 2, and 3) within the regression model. Subgroup analyses were conducted to test the consistency of the association across different strata, including sex, age (<60 vs. ≥60 years), hypertension, atrial fibrillation, diabetes, coronary heart disease, and intravenous thrombolysis. Interaction terms were introduced into the regression models to evaluate potential effect modification, and significance of these interactions was tested. A sensitivity analysis was performed by treating extreme HRR values as missing and re-running the multiple imputation and multivariable regression analysis to ensure the results were not driven by outliers. To validate that the observed HRR-SAP association was not simply driven by one of its individual components, we performed comparative association analyses. Three separate logistic regression models were constructed, each adjusted for the same covariates: (1) Model A with HRR as the exposure; (2) Model B with hemoglobin (per 10 g/L) as the exposure; (3) Model C with RDW (per 1%) as the exposure. Model fit was compared using Akaike Information Criterion (AIC) and Bayesian Information Criterion (BIC), with lower values indicating better fit. C-statistics (area under the ROC curve) were also calculated to assess overall model discrimination. DeLong’s test was used to compare C-statistics between models. All statistical analyses were performed using R software(version 4.4.2; R Foundation for Statistical Computing, Vienna, Austria) with the following packages: mgcv (v1.9-3) for generalized additive models, mice (v3.19.0) for multiple imputation, and pROC (v1.19.0.1) for C-statistic calculation and DeLong’s test. A two-sided P-value < 0.05 was considered statistically significant.

## 3 Results

### 3.1 Baseline characteristics

A total of 1051 patients with acute ischemic stroke were included in the final analysis. Among them, 94 (8.94%) developed SAP during hospitalization. The detailed baseline characteristics of the study population, stratified by the occurrence of SAP, are presented in **Table 1**. Compared to patients in the non-SAP group, those who developed SAP were significantly older (61.55 ± 12.54 vs. 69.02 ± 14.09 years, p < 0.01), had a longer hospital stay (9.66 ± 5.12 vs. 13.19 ± 6.7 days, p < 0.01), and exhibited a higher prevalence of comorbidities such as coronary heart disease (7.42% vs. 17.02%, p < 0.01) and atrial fibrillation (4.91% vs. 19.15%, p < 0.01). Regarding laboratory parameters at admission, the SAP group showed significantly lower levels of hemoglobin (132.98 ± 16.65 vs. 139.43 ± 17.44 g/L, p < 0.01) and HRR (10.78 ± 1.67 vs. 10.02 ± 1.62, p < 0.01). Conversely, the RDW was significantly higher in the SAP group (13.05 ± 1.13% vs. 13.39 ± 1.08%, p < 0.01). Patients who received venous thrombolysis were also more likely to develop SAP (12.54% vs. 29.79%, p < 0.01).

**Table 1:** Baseline characteristics of patients with and without SAP.

Variable names	Level	Overall	Non-SAP	SAP	*p*	SMD
n		1051	957	94		
Age, years		62.22 ± 12.86	61.55 ± 12.54	69.02 ± 14.09	<0.01	0.56
Length of hospital stay, days		9.98 ± 5.38	9.66 ± 5.12	13.19 ± 6.70	<0.01	0.59
Creatinine(umol/l)		86.54 ± 57.31	86.5 ± 58.79	86.93 ± 39.52	0.95	0.01
Blood Glucose(mmol/L)		8.43 ± 4.54	8.46 ± 4.61	8.14 ± 3.83	0.52	0.08
Uric Acid(umol/l)		364.33 ± 109.74	365.3 ± 108.05	354.47 ± 125.88	0.36	0.09
TG(mmol/L)		1.74 ± 1.27	1.76 ± 1.27	1.56 ± 1.23	0.14	0.16
HDL(mmol/L)		1.09 ± 0.29	1.09 ± 0.28	1.15 ± 0.37	0.03	0.21
LDL(mmol/L)		3.11 ± 0.98	3.11 ± 0.96	3.08 ± 1.12	0.72	0.04
Hemoglobin(g/L)		138.85 ± 17.46	139.43 ± 17.44	132.98 ± 16.65	<0.01	0.38
Rdw(%)		13.08 ± 1.13	13.05 ± 1.13	13.39 ± 1.08	<0.01	0.31
TC(mmol/L)		4.68 ± 1.1	4.68 ± 1.09	4.63 ± 1.25	0.70	0.04
White Blood Cells(WBC),10^9/L		8.41 ± 2.87	8.26 ± 2.75	9.85 ± 3.63	<0.01	0.49
HRR		10.71 ± 1.68	10.78 ± 1.67	10.02 ± 1.62	<0.01	0.46
Gender (%)	male	724 (68.89)	659 (68.86)	65 (69.15)	1.00	0.01
	female	327 (31.11)	298 (31.14)	29 (30.85)		
Diabetes (%)	No	668 (63.56)	604 (63.11)	64 (68.09)	0.40	0.10
	Yes	383 (36.44)	353 (36.89)	30 (31.91)		
Coronary Heart Disease(%)	No	964 (91.72)	886 (92.58)	78 (82.98)	<0.01	0.30
	Yes	87 (8.28)	71 (7.42)	16 (17.02)		
Atrial Fibrillation (%)	No	986 (93.82)	910 (95.09)	76 (80.85)	<0.01	0.45
	Yes	65 (6.18)	47 (4.91)	18 (19.15)		
Hypertension (%)	No	161 (15.32)	150 (15.67)	11 (11.70)	0.38	0.12
	Yes	890 (84.68)	807 (84.33)	83 (88.30)		
Venous thrombolysis(%)	No	903 (85.92)	837 (87.46)	66 (70.21)	<0.01	0.43
	Yes	148 (14.08)	120 (12.54)	28 (29.79)		

TG, Triglyceride; TC, Total cholesterol; LDL-C,Low-density lipoprotein cholesterol; HDL-C, High-density lipoprotein cholesterol; RDW, Red blood cell distribution width; HRR, Hemoglobin-to-red blood cell distribution width ratio; SAP, Stroke-associated pneumonia.

### 3.2 Association between HRR and SAP risk

**Figure 2:**
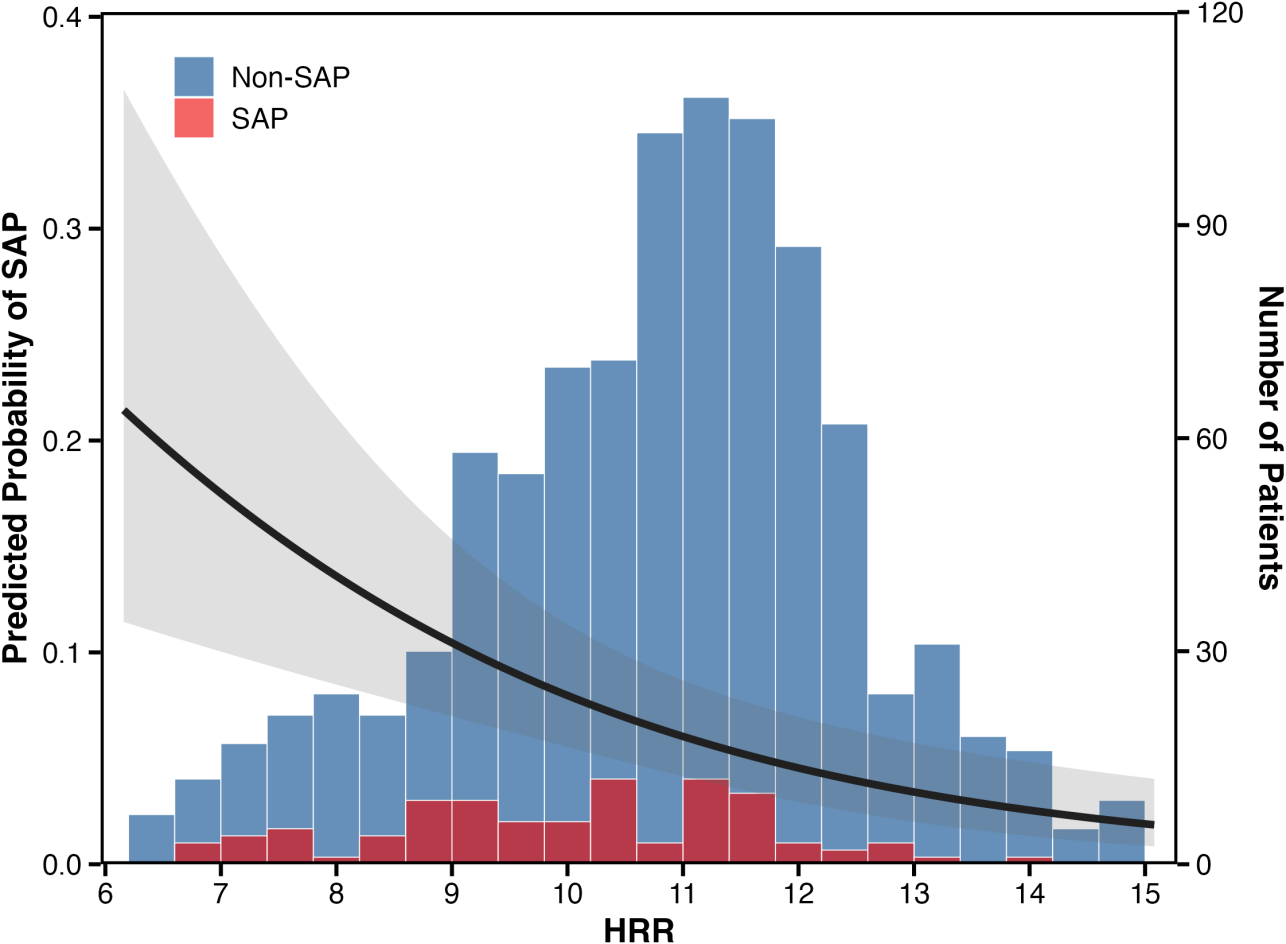
The relationship between HRR and the predicted probability of SAP. The solid line represents the smooth curve fitted by a generalized additive model (GAM), and the shaded band represents the 95% confidence interval. The histogram displays the distribution of HRR values stratified by SAP status (blue: Non-SAP, n=957; red: SAP, n=94). All adjusted for: sex, age, diabetes, hypertension, atrial fibrillation, coronary heart disease, intravenous thrombolysis, triglycerides, total cholesterol, LDL-C, HDL-C, and white blood cells.

Before conducting regression analysis, we used a generalized additive model (GAM) with smoothing splines to visualize the shape of the association between HRR and SAP risk (**Figure 2**). The smooth curve revealed a linear negative relationship across the observed range of HRR values, with no evidence of threshold effects or non-linearity. The 95% confidence band remained relatively narrow in the middle range of HRR but widened at the extremes, reflecting fewer observations in those regions. This linear pattern justified treating HRR as a continuous variable in the subsequent regression models.

### 3.3 Multivariable logistic regression analysis

**Table 2** details the findings of the multivariable logistic regression. The unadjusted model showed that higher HRR was linked to reduced SAP risk. This inverse relationship persisted after sequential adjustment for covariates in Model 1 and Model 2. In the fully adjusted model (Model 2), each unit increase in HRR was associated with a 25% reduction in SAP odds (OR = 0.75, 95% CI: 0.64, 0.87). When HRR was analyzed as a categorical variable, patients in the top tertile (T3) had lower SAP risk compared to those in the bottom tertile (T1) (Adjusted OR = 0.34, 95% CI: 0.17, 0.66). A dose-response pattern was confirmed by a significant test for trend (p for trend < 0.001).

**Table 2:** Relationship between HRR and risk of SAP across sequential adjustment models.

Variable	Crude model	*P*	Model 1	*P*	Model 2	*P*
	95% CI		95% CI		95% CI	
HRR	0.77 (0.68,0.87)	<0.001	0.80 (0.70,0.92)	<0.01	0.75 (0.64,0.87)	<0.001
HRR Tertiles						
T1	ref		ref		ref	
T2	0.68 (0.42,1.09)	0.11	0.72 (0.43,1.18)	0.19	0.64 (0.37,1.10)	0.11
T3	0.35 (0.19,0.61)	<0.001	0.44 (0.23,0.81)	<0.05	0.34 (0.17,0.66)	<0.01
*p* for trend		<0.001		<0.01		<0.01

Crude model: no variables are adjusted.

Model 1 adjust for: sex and age.

Model 2 adjust for: sex and age; diabetes, hypertension, coronary heart disease, atrial fibrillation; venous thrombolysis; TG, LDL-C and TC, HDL-C, WBC.

TG, Triglyceride; TC, Total cholesterol; LDL-C, Low-density lipoprotein cholesterol; HDL-C, High-density lipoprotein cholesterol; WBC, White blood cells; SAP, stroke-associated pneumonia.

### 3.4 Comparative association analysis

To validate the independent value of HRR as a composite marker, we compared its association with SAP to those of its individual components (**Table 3**). In fully adjusted models, HRR (OR = 0.75, 95% CI: 0.65-0.87, p < 0.001) showed the strongest association with SAP risk. Hemoglobin (per 10 g/L increase) also demonstrated a significant inverse association (OR = 0.77, 95% CI: 0.66-0.89, p < 0.001), while RDW (per 1% increase) showed a positive association (OR = 1.21, 95% CI: 1.02-1.45, p = 0.031).

**Table 3:** Comparative association analysis of HRR versus individual components.

Model	Exposure variable	OR (95% CI)	P-value	AIC	BIC	C-statistic (95% CI)
Model A	HRR (per 1-unit)	0.75 (0.65-0.87)	<0.001	567.5	632.0	0.784 (0.737-0.832)
Model B	Hemoglobin (per 10 g/L)	0.77 (0.66-0.89)	<0.001	570.2	634.6	0.777 (0.729-0.826)
Model C	RDW (per 1%)	1.21 (1.02-1.45)	0.031	577.9	642.3	0.764 (0.713-0.815)

All models adjusted for: sex, age, diabetes, hypertension, coronary heart disease, atrial fibrillation, triglycerides, total cholesterol, LDL-C, HDL-C, and white blood cells.

OR, odds ratio; CI, confidence interval; AIC, Akaike Information Criterion; BIC, Bayesian Information Criterion; HRR, hemoglobin-to-red blood cell distribution width ratio; RDW, red blood cell distribution width.

Model fit comparison revealed that the HRR model had the lowest AIC (567.5) and BIC (632.0), compared to the hemoglobin model (AIC = 570.2, BIC = 634.6; ΔAIC = +2.7) and the RDW model (AIC = 577.9, BIC = 642.3; ΔAIC = +10.4). The HRR model also demonstrated the highest C-statistic (0.784, 95% CI: 0.737-0.832), which was significantly better than the RDW model (0.764, 95% CI: 0.713-0.815; p = 0.03) but similar to the hemoglobin model (0.777, 95% CI: 0.729-0.826; p = 0.26).

### 3.5 Subgroup analyses

**Figure 3:**
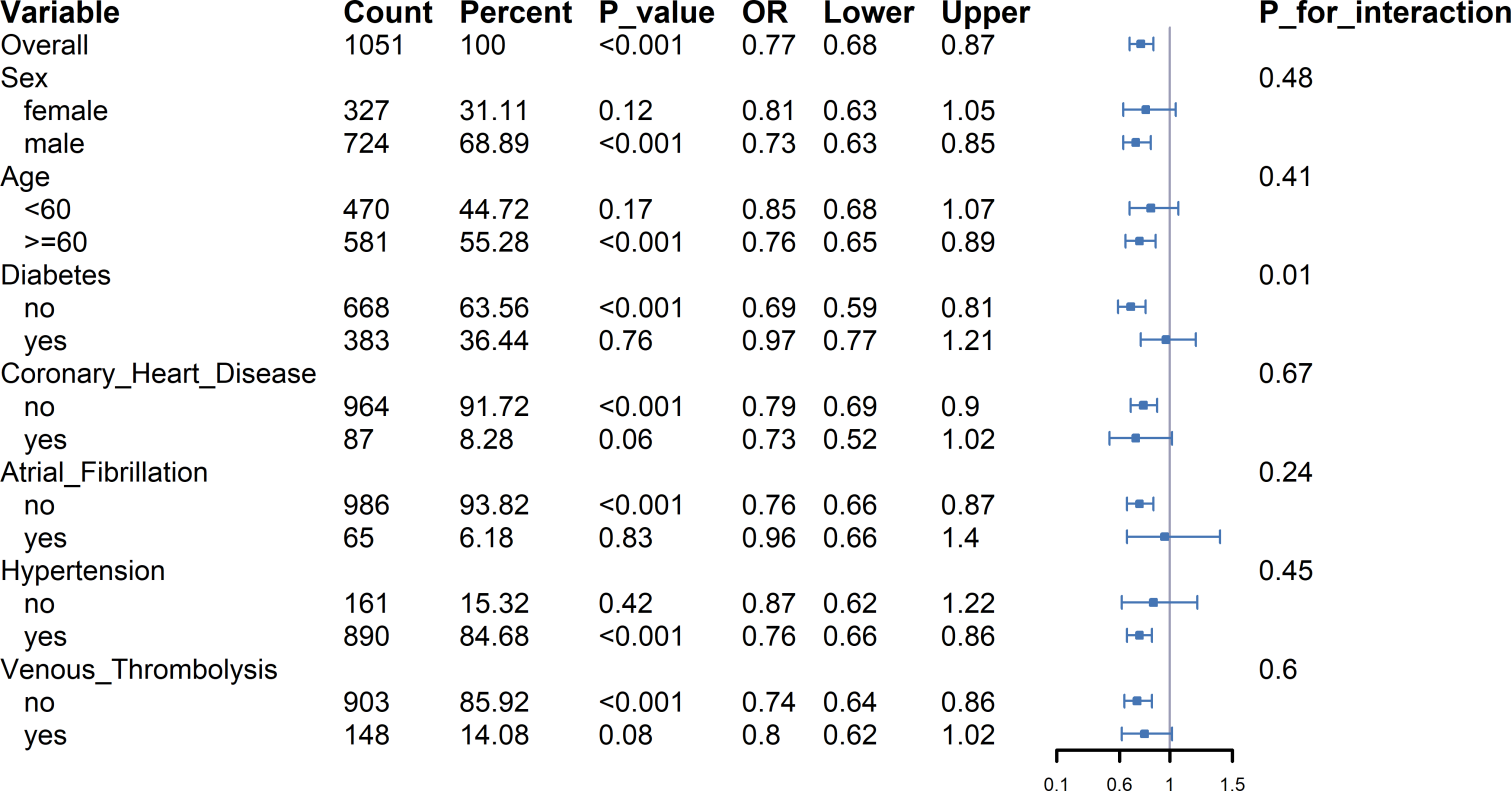
Forest plot of subgroup analyses for the association between HRR (as a continuous variable) and risk of SAP.

The results of the subgroup analysis are presented in **Figure 3**. The inverse association between HRR and SAP risk was consistent across most prespecified subgroups. No significant interactions were found for sex (p = 0.48), age (p = 0.41), coronary heart disease (p = 0.67), atrial fibrillation (p = 0.24), hypertension (p = 0.45), or venous thrombolysis (p = 0.6).

A significant interaction was detected between HRR and diabetes status (p for interaction = 0.01). In non-diabetic patients (n=668, 64 SAP events), higher HRR was associated with reduced SAP risk (OR = 0.69; 95% CI: 0.59, 0.81; p < 0.001). In diabetic patients (n=383, 30 SAP events), this association was absent (OR = 0.97; 95% CI: 0.77, 1.21; p = 0.76). This was the only significant effect modification observed among all tested subgroups.

### 3.6 Sensitivity analyses

To assess the robustness of our findings, we performed sensitivity analyses by treating extreme HRR values (identified by box plots) as missing and re-running the multiple imputation and regression analysis. The results remained consistent with the primary analysis. In the fully adjusted model, HRR was inversely associated with SAP risk (OR = 0.76; 95% CI: 0.66, 0.88; p < 0.001), with effect estimates similar to those observed in the main analysis (OR = 0.75). The direction, magnitude, and statistical significance of the association were preserved across all three models (**Table 4**), indicating that the observed association was not driven by outliers.

**Table 4:** Relationship between HRR and risk of SAP across sequential adjustment models.

Variable	Crude model	*P*	Model 1	*P*	Model 2	*P*
	95% CI		95% CI		95% CI	
HRR	0.77 (0.69,0.87)	<0.001	0.80 (0.70,0.91)	<0.001	0.76 (0.66,0.88)	<0.001

Crude model: no variables are adjusted.

Model 1 adjust for: sex and age.

Model 2 adjust for: sex and age; diabetes, hypertension, coronary heart disease, atrial fibrillation; venous thrombolysis; TG, LDL-C and TC, HDL-C, WBC.

TG, Triglyceride; TC, Total cholesterol; LDL-C, Low-density lipoprotein cholesterol; HDL-C, High-density lipoprotein cholesterol; WBC, White blood cells; SAP, stroke-associated pneumonia.

## 4 Discussion

In this retrospective study, we investigated the association between admission HRR and early-onset SAP risk in AIS patients. Our main finding is that lower admission HRR was associated with increased SAP risk. We observed a stable, linear negative association between HRR and SAP risk. This inverse association remained statistically significant (adjusted OR = 0.75; 95% CI: 0.64, 0.87) after adjusting for available demographic and clinical confounders, indicating that each unit increase in HRR was associated with approximately 25% lower odds of SAP. This association was consistent across most predefined clinical subgroups and remained robust in sensitivity analyses. These results suggest that HRR may be a useful, cost-effective biomarker for SAP risk assessment.

A key finding is the significant interaction between HRR and diabetes status in predicting SAP risk (p for interaction = 0.01). The inverse association was evident in non-diabetic patients (OR = 0.69; 95% CI: 0.59, 0.81; p < 0.001) but absent in diabetic patients (OR = 0.97; 95% CI: 0.77, 1.21; p = 0.76). This finding is biologically plausible. Diabetes is characterized by chronic low-grade inflammation, impaired neutrophil function, and endothelial dysfunction. These diabetes-specific pathways may attenuate the prognostic utility of HRR. Diabetic patients often have elevated baseline RDW due to chronic inflammation and oxidative stress, which may reduce the discriminative ability of HRR. The immunosuppressive effects of hyperglycemia may also overwhelm the signals captured by HRR, and diabetes-related microvascular changes may alter the relationship between hematological parameters and infection susceptibility. Notably, the diabetic subgroup is inherently heterogeneous. Variations in glycemic control (HbA1c), diabetes duration, and the presence of microvascular complications (retinopathy, nephropathy) may differentially influence the HRR-SAP relationship. For instance, poorly controlled diabetes with chronically elevated RDW may diminish HRR’s discriminative ability, while well-controlled or early-stage diabetic patients might still exhibit an association similar to non-diabetic individuals. Future studies stratifying by these diabetes-specific characteristics are needed to test this hypothesis.

This phenomenon—where diabetes attenuates the predictive value of biomarkers—has been observed in other clinical contexts. In the Strong Heart Study, CRP predicted cardiovascular events in non-diabetic individuals, but this association was weakened in diabetic patients after accounting for other inflammatory markers (13). Similarly, a study on NLR in renal cell carcinoma reported that diabetes attenuated the prognostic value of NLR (25). Our finding extends this pattern to the SAP prediction domain, suggesting that the chronic inflammatory state of diabetes may reduce the discriminative ability of inflammation-related biomarkers across different clinical outcomes.

Hatzitolios et al. reported that type 2 diabetes was not independently associated with pneumonia incidence after acute ischemic stroke (26). Our finding complements this observation: while diabetes may not directly increase SAP risk, it modifies how other risk factors—specifically HRR—operate. This distinction between direct effects and effect modification suggests that risk stratification strategies may need to account for diabetes status.

Hb is an indicator of oxygen-carrying capacity and nutritional status. Low Hb levels, or anemia, are associated with poor functional outcomes and increased mortality in stroke patients (27, 28). Reduced Hb can exacerbate cerebral and systemic hypoxia, impairing immune cell function and host defense, thereby increasing infection susceptibility (29–31). RDW is a marker of systemic inflammation and oxidative stress (32). Elevated RDW, reflecting high variability in erythrocyte size (anisocytosis), has been linked to adverse prognoses in various critical conditions, including stroke and sepsis (33–35). The strength of HRR may lie in its integration of these two pathophysiological dimensions. A low HRR value represents a vulnerable patient profile: diminished physiological reserve (low Hb) combined with heightened systemic stress and inflammation (high RDW). This combined state of fragility may render patients more susceptible to post-stroke immunosuppression and subsequent bacterial infection. Our comparative analyses demonstrate that HRR integrates complementary pathophysiological information. While the HRR model showed similar discrimination to the hemoglobin model (C-statistic: 0.784 vs 0.777, p = 0.26), it demonstrated superior model fit (lower AIC/BIC) and significantly better discrimination than the RDW model (ΔC-statistic = 0.020, p = 0.03). This suggests that HRR captures both oxygen-carrying capacity (reflected by hemoglobin) and systemic inflammation (reflected by RDW), with the ratio format potentially offering a more balanced integration of these two dimensions.

From a clinical applicability perspective, HRR offers several practical advantages. First, it is derived from routine complete blood count testing performed at admission, requiring no additional cost or specialized equipment. Second, as a continuous marker showing a linear association with SAP risk, HRR does not require arbitrary cutoff values for clinical interpretation. Third, patients with lower HRR values (particularly those below the median of 10.9 in our cohort, especially non-diabetic patients) may benefit from enhanced preventive interventions, including more frequent swallowing assessments, intensified oral hygiene protocols, and closer respiratory surveillance. The integration of HRR into existing SAP prediction scores such as A2DS2 or ISAN represents a promising direction for future research. As a continuous variable derived from routine testing, HRR could potentially improve risk stratification accuracy when combined with clinical factors. However, prospective validation studies are needed to determine the optimal approach for incorporating HRR into clinical decision-making algorithms and to establish whether HRR-guided interventions can reduce SAP incidence in real-world practice.

Our study has several limitations. Due to the retrospective design, we were unable to adjust for several key prognostic factors, most notably stroke severity (NIHSS score) and swallowing function (dysphagia assessment), mechanical ventilation status, ICU admission, and nasogastric tube use. As these factors are powerful predictors of SAP and are likely correlated with systemic stress markers like HRR, the true independence of the observed association remains unclear. Although the association persisted after adjusting for available confounders, residual confounding from these unmeasured variables is possible and warrants cautious interpretation of the effect size. Other potentially informative variables were also unavailable, including hematocrit, RBC count, MCV, nutritional and iron-status markers (ferritin, transferrin), and antibiotic prophylaxis data. The interaction between HRR and diabetes status was statistically significant; however, the stratified sample sizes were relatively small (30 SAP events in diabetic patients), which may limit the precision of subgroup-specific estimates. This interaction finding should be considered hypothesis-generating and requires validation in larger cohorts. Moreover, the absence of HbA1c, diabetes duration, and microvascular complication data precluded further characterization of heterogeneity within the diabetic subgroup. This study explored associations rather than developing a prediction model. The incremental predictive value of HRR over its individual components for clinical risk stratification requires validation in independent cohorts with comprehensive clinical data (NIHSS, dysphagia assessment) and comparison with established SAP prediction scores (e.g., A2DS2, ISAN). The retrospective, single-center design may also limit generalizability, and retrospective ascertainment of SAP may be subject to information bias, and precise timing data for SAP diagnosis (e.g., median time from admission to diagnosis) were not available to further characterize the temporal relationship between HRR measurement and SAP onset. HRR was measured at a single time point upon admission; serial assessments could provide dynamic information on hematological changes during hospitalization. Future prospective, multi-center studies are needed to validate these findings and to quantify HRR’s additive clinical value by incorporating it into comprehensive models that include NIHSS and dysphagia assessments.

## 5 Conclusion

In conclusion, admission HRR is inversely associated with SAP risk in AIS patients. This association is modified by diabetes status, with the inverse association present in non-diabetic patients (OR = 0.69) but absent in diabetic patients (OR = 0.97). This finding suggests that SAP risk stratification may need to account for diabetes status. Future prospective studies are needed to validate these findings and explore diabetes-specific risk prediction approaches.

## Data Availability

The datasets presented in this article are not readily available because the data contain sensitive patient information from a retrospective clinical study, and full public deposition is not feasible due to patient privacy and institutional ethical restrictions. Requests to access the datasets should be directed to the corresponding author, Xiaoqiang Li (liboleq@163.com).
